# *Mycobacterium tuberculosis* genotypes in an ethnically diverse area with millions of pilgrims and thousands of immigrants

**DOI:** 10.1186/s12879-023-08325-6

**Published:** 2023-05-25

**Authors:** Mostafa Naseri-Nezhad, Mahla Asadian, Mohammad Khalifeh Gholi, Mehdi Yaseri, Masoumeh Douraghi

**Affiliations:** 1 Division of Microbiology, Department of Pathobiology, School of Public Health, Tehran University of Medical Sciences, Tehran, Iran; 2 Department of Microbiology and Immunology, Faculty of Medicine, Qom University of Medical Sciences, Qom, Iran; 3 Department of Epidemiology and Biostatistics, School of Public Health, Tehran University of Medical Sciences, Tehran, Iran

**Keywords:** Immigration, MIRU-VNTR, Qom, Tuberculosis

## Abstract

**Background:**

Immigration is considered as a risk factor of tuberculosis (TB). Qom province receives millions of pilgrims and significant numbers of immigrants each year. Most of the immigrants to Qom, arrive from neighboring TB-endemic countries. This study aimed to identify the current circulating *Mycobacterium tuberculosis* genotypes in Qom province using 24-locus MIRU-VNTR genotyping.

**Methods:**

Eighty six *M. tuberculosis* isolates were collected during 2018–2022 from patients referring to Qom TB reference laboratory. The DNA of isolates was extracted and followed by 24 loci MIRU-VNTR genotyping which performed using the web tools available on MIRU-VNTR*plus*.

**Results:**

Of 86 isolates, 39 (45.3%) were of Delhi/CAS genotype, 24 (27.9%) of NEW-1, 6 (7%) of LAM, 6 (7%) of Beijing, 2 (2.3%) of UgandaII, 2 (2.3%) of EAI, 1 of S (1.2%) and 6 (7%) did not match with profiles present in MIRUVNTR*plus* database.

**Conclusions:**

About half of the isolates belong to Afghan immigrants; which warns health policy makers about the future situation of TB in Qom. Also, the similarity of Afghan and Iranian genotypes provides evidence that immigrants partake in the circulation of *M. tuberculosis*. This study underpin the studies about the circulating *M. tuberculosis* genotypes, their geographical distribution, the association of TB risk factors with these genotypes and the impact of immigration on the situation of TB in Qom province.

**Supplementary Information:**

The online version contains supplementary material available at 10.1186/s12879-023-08325-6.

## Introduction

Immigrants moving from a high-incidence areas to a low-incidence areas can have substantially higher tuberculosis (TB) rates than natives due to more contact with *Mycobacterium tuberculosis* (*M. tuberculosis*) in their countries of origin [1]. Latent tuberculosis infection (LTBI) in immigrants can turn into active disease because of the unfavorable socio-economic conditions that they might face during or after immigration. The understanding of the underlying reasons for LTBI reactivation is incomplete, but it does include bacterial, host and environmental factors. Some of the risk factors in human might be relevant to infection with HIV, history of imprisonment, drug addiction, living in crowded houses and poverty. The activation of TB and its’ transmission can become a risk for natives and immigrants themselves and also their close contacts. Several studies have demonstrated high incidence rates of TB among immigrants who immigrated to low-incidence countries during the early years post-immigration [1–8].

Tuberculosis incidence rate in Iran is 7.36 per 100,000 inhabitants [9]. Iran is bordered by TB-endemic countries including Afghanistan and Pakistan and by countries with higher TB rates than Iran, including Azerbaijan and Turkey [10]. Significant number of Afghan immigrants come to Iran because of its proximity to their country of origin and due to the existing similarities in religion and culture. Moreover, some patients from Azerbaijan visit health centers in the North-West of Iran, for the low-cost or free-of-charge TB treatment [11]. Qom province is considered as one of the first destinations for immigration in Iran. It is located 140 km to the south of Tehran. Apart from proximity to the capital, every year Qom attracts millions of pilgrims and thousands of immigrants because of being a remarkable center of teaching the Islamic directives and by the existence of the holy shrine. Also, Qom is an intersection area that links the different provinces of the country, which drives even more people that normally tend to visit the holy shrine on the way. According to the last 2016 census, Qom’s population was 2,431,122 and more than 240,000 people of the province’s population are immigrants that mostly come from Afghanistan, Iraq and Pakistan. In 2016, Afghan immigrants numbers were 96,367 (40%) of all immigrants, Iraqi 8,365 (3.48%), Pakistani 6,543 (2.7%) and more than 8,700 (3.6%) were from other countries [12]. Normally, immigrants from the same nationality tend to live close to each other which creates a community of a specific nationality over the time. As noted, most of the immigrants come to Qom from countries where TB incidence rate are relatively high [10]. Although TB incidence rate in Qom is 8.8 per 100,000 inhabitants and is close to the country’s average [9], but due to its’ unique conditions, Qom was selected for this study. There is no sufficient information about the circulating *M. tuberculosis* genotypes in Qom. Hence, this study aimed to identify the current circulating *M. tuberculosis* genotypes in Qom using 24-locus MIRU-VNTR typing.

## Materials and methods

### Study setting

During July 2018 to March 2022, only TB cases that had a positive culture for *Mycobacterium tuberculosis* complex (MTBC) were included in this study. A part of this study was performed at TB reference laboratory of Qom province which provides free TB diagnosis services for both urban and rural areas of Qom. Furthermore, the typing was done in Tehran University of Medical Sciences. The study was approved by the ethics committee of Tehran University of Medical Sciences, Tehran, Iran (IR.TUMS.SPH.REC.1399.241).

### Isolate collection

The clinical specimens of TB suspected cases who were referred to TB reference laboratory of Qom province were decontaminated using Petroff method [13] and for culture, Lowenstein-Jensen (LJ) medium was used. To warrant the primary isolation of *Mycobacterium tuberculosis* (MTB), phenotypic characteristics such as colony morphology and pigment production were assessed. The genomic DNA of isolates was extracted by Cetyl trimethylammonium bromide (CTAB) method [14]. For the identification of MTB, a PCR using region of difference 1 (RD-1) primer [15] was performed and a 150-bp amplicon was observed on the agarose gel.

### MIRU-VNTR typing

The 24 loci MIRU-VNTR typing was performed using the standard protocol described by Supply et al. [16]. PCR and gel electrophoresis was done and the size of PCR products was compared to the standard allelic table of MIRU-VNTR [16]. The produced 24 number numerical types were analyzed with the web based tools at: www.miru-vntrplus.org.

### Statistical analysis

The variables considered in this study were: age, gender, nationality, place of residence, occupation, number of family members, history of contact with TB patient, diabetes, infection with HIV, history of imprisonment, history of drug abuse, marital status, education level, final outcome of TB, and history of TB in the family. Statistical analysis was done using IBM SPSS Statistics 26.00 software (IBM Corp., USA) and *P*-value of less than 0.05 was considered statistically significant [17, 18]. Placing the patients in the middle and low income levels was done after comparing the known zones income level reported by Management and Planning Organization of Qom [12] with the Iran’s current poverty line stated by the government each year.

### The calculation of allelic diversity (h)

Allelic diversity (h) of each locus was calculated using Hunter-Gaston discriminatory Index (HGDI) formula [19]. Values of HGDI vary between 0.00 and 1.00. Loci with h > 0.6 were regarded as loci with high allelic diversity, 0.3 ≤ h ≤ 0.6 were regarded as moderately diverse and h < 0.3 regarded as loci with low levels of allelic variability.

## Results

### Demographic information

Of patients who had a positive culture for MTB and included in this study, 85% lived in urban areas and 13% lived in rural areas. The 42 (48.8%) of the isolates examined belong to Qom’s native people and the remaining 41 (47.7%) belong to Afghan immigrants, 2 (2.3%) Indian immigrants and 1 (1.3%) Pakistani immigrant. With regard to gender of TB cases in this study, males were 45 (52.3%) and females 41 (47.7%) and the average age ± SD of patients was 55 ± 36 years.

More than half of patients 38 (53.5%) were housewives and the others were laborers 14 (19.7%) or unemployed people 8 (11.3%). The remaining were: clergies 4 (5.6%), self-employed 3 (4.2%), taxi drivers 2 (2.8%) and 2 (2.8%) retired teachers. Also, the unemployment in Afghan immigrants was almost two fold of Iranians and Afghan laborers numbers was twice as many as Iranians.

A considerable number of patients had low levels of education including illiterate patients 45 (64.3%), patients with primary school degree 10 (14.3%), middle school degree 3 (4.3%), high school diploma 5 (7.1%), B.Sc. degree 5 (7.1%), M.Sc. degree 2 (2.9%) and the level of education of the remaining of the patients was unknown. Table 1 shows a comparison of demographic factors between native and immigrant patients.

**Table 1:** Demographics and risk factors in natives *vs*. immigrant patients

Variable	Type of variable	Native (%)	Immigrant (%)
Gender	Man	56.4	51.4
	Woman	43.6	48.6
Marital status	Single	10.3	22.9
	Married	41	54.3
	Divorced	0	0
	Widow/er	23.1	20
	No data available	25.6	9.2
History of contact with TB patient	Negative	69	75.7
HIV	Positive	31	24.3
	Negative	100	100
Diabetes	Positive	0	0
	Negative	69.3	90.6
History of imprisonment	Positive	30.7	9.4
	Negative	96.2	98.4
Drug addiction	Positive	3.8	0
	Negative	96.2	100
Smear microscopy result	Positive	8.3	0
	1–9 bacili in every microscopic field	2.6	2.5
	+ 1	10.3	8.6
	+ 2	5.1	14.3
	+ 3	5.1	5.7
	Smear negative	77	71.4
Place of residence	Urban areas	87.8	84.8
	Rural areas	12.2	15.2
Final disease outcome	Cured of TB	3.77	90.8
	Death	22.5	3.3
History of TB in family	Negative	86.2	94
	Positive	13.8	6
Level of education	Illiterate	35.9	77.1
	Primary school degree	17.9	2.9
	Middle school degree	2.6	5.7
	High school diploma	5.1	4.8
	Bachelor’s degree	7.7	2.9
	Master’s degree	0	3.8
	PhD degree	0	0
	No data available	30.8	4.8
Patient’s job	Jobless	7.7	14.3
	Laborer	10.3	22.9
	housewife	38.5	51.4
	Clergy	2.6	8.7
	Retired	5.2	0
	Taxi driver	5.1	0
	No data available	30.8	7.2

### Risk factors of TB

Regarding to TB risk factors assessed, diabetes in 11 (15.9%), history of imprisonment in 1 (1.4%) and drug addiction in 1 (1.4%) case were identified. None of the patients had HIV infection. Furthermore, reviewing patients’ data revealed that 21 (24.4%) had a previous contact with a TB patient.

*Identified Mycobacterium tuberculosis genotypes*.

**Fig. 1:**
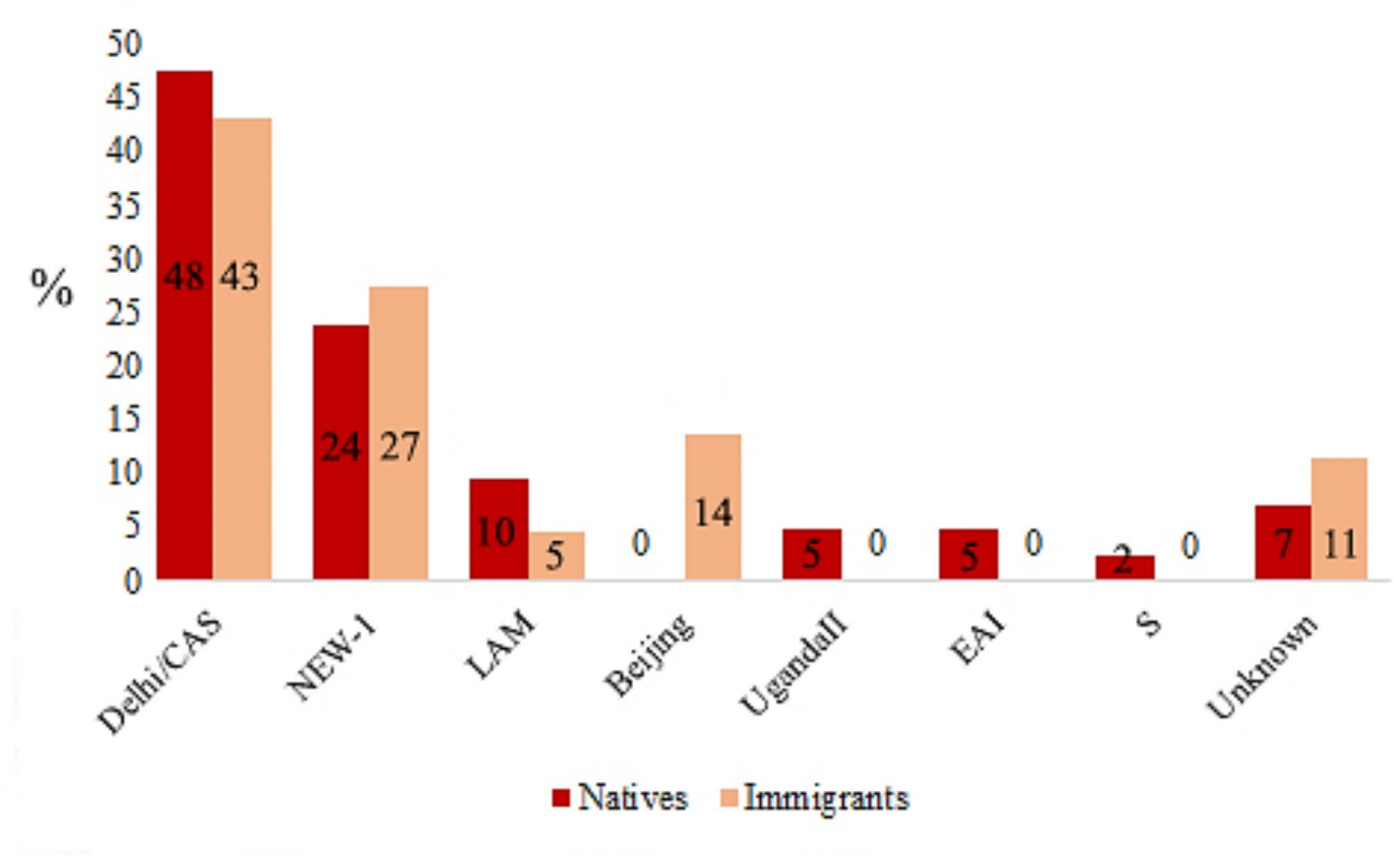
*Mycobacterium tuberculosis* genotypes identified in the natives and immigrants

Of 86 MTB isolates tested in Qom province, 39 (45.3%) isolates were of Delhi/CAS genotype, 24 (29.7%) of NEW-1, 6 (7.0%) of LAM, 6 (7.0%) of Beijing, 2 (2.3%) of UgandaII, 2 (2.3%) of EAI, 1 isolate of S (1.2%) and 6 (7.0%) isolates did not match with profiles present in MIRUVNTR*plus* database (Table S1). Beijing genotype was only identified among Afghan immigrants and LAM, EAI, UgandaII and S genotypes were only identified among natives. Figure 1 shows the percentages of identified genotypes among natives and immigrants.

### Distribution of genotypes over the time

**Fig. 2:**
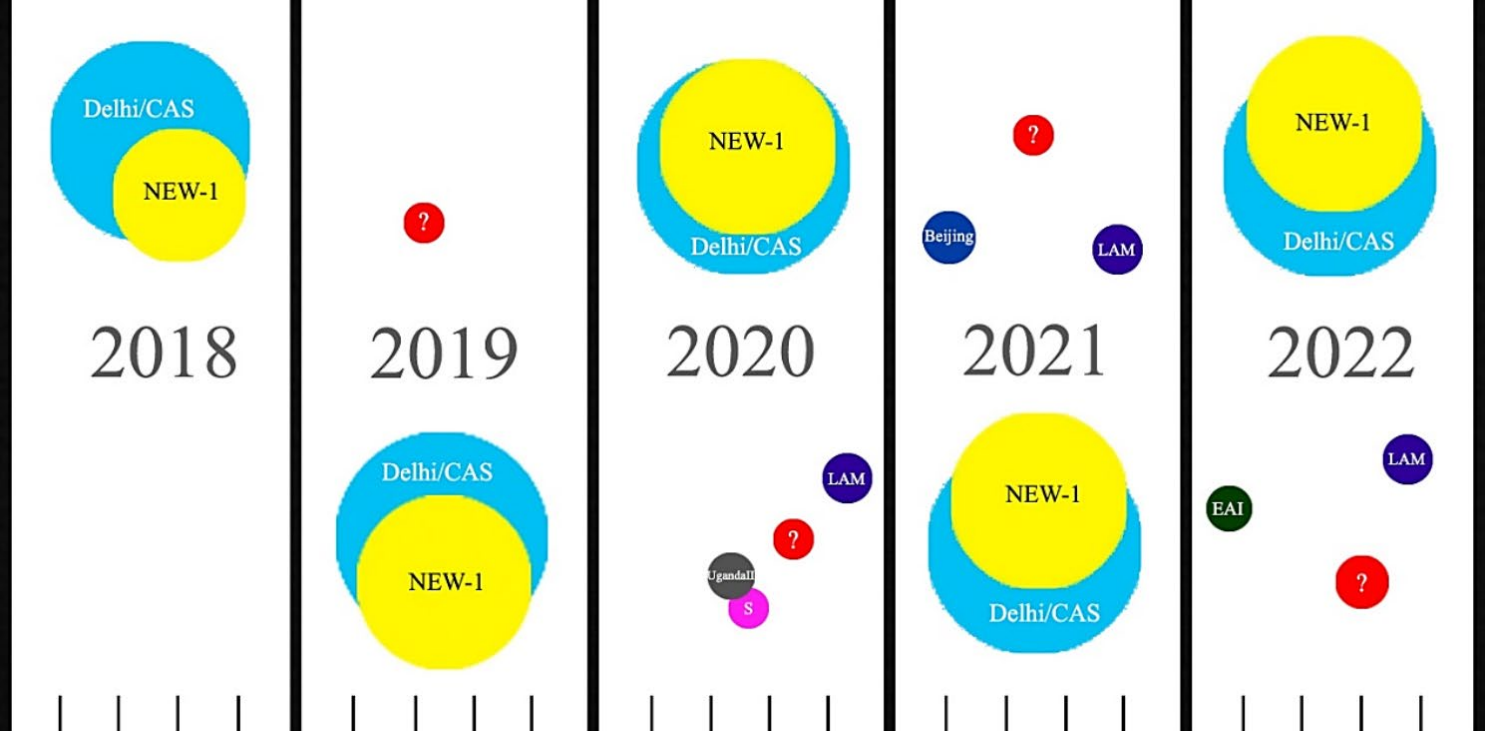
Different identified *Mycobacterium tuberculosis* genotypes in Qom in the period of 5 years. Different color circles indicate different genotypes (Cyan: Delhi/CAS, yellow: NEW-1, purple: LAM, blue: Beijing, grey: UgandaII, green: EAI, Pink: S and red shows isolates that did not match with profiles present in MIRUVNTR*plus* database.) The size of color circles show the approximate genotype frequency. The bottom horizontal line show a simple time line that shows the time of identification of a genotype

During the period of collection of isolates, the genotypic distribution was not the same. From the beginning of 2018 to the end of 2019, Delhi/CAS and NEW-1 were the only genotypes identified. From mid-2020, other genotypes such as S, UgandaII were identified while LAM was firstly identified in the end of 2020. While Beijing was identified in early 2021, EAI was identified in the beginning of 2022 (Fig. 2). A notable point is that the two genotypes of Delhi/CAS and NEW-1 remained as the dominant genotypes over the period of 5 years.

### The geographic distribution of MTB genotypes

**Fig. 3:**
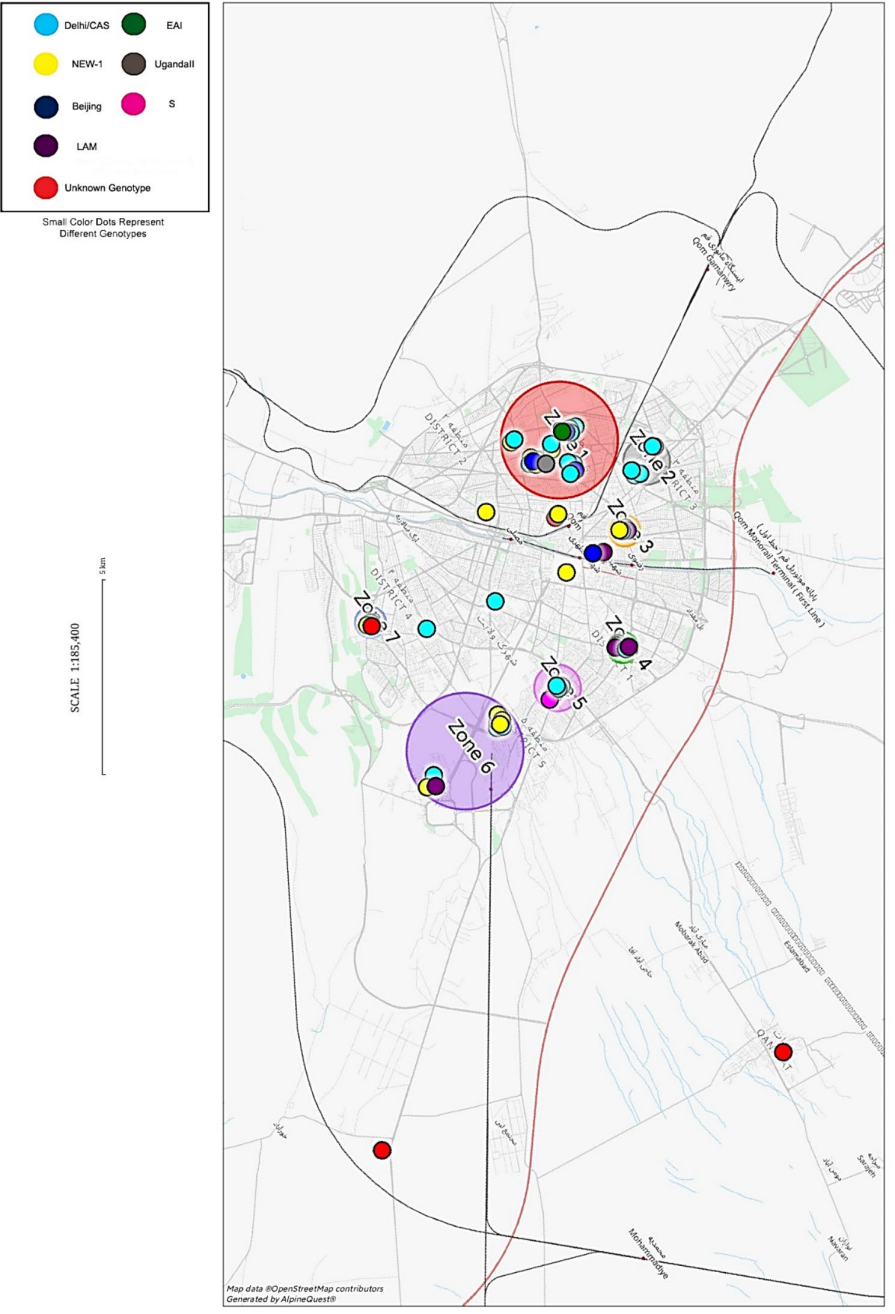
*Mycobacterium tuberculosis* genotypes geographic distribution in Qom (Map is generated by AlpineQuest). Color dots represent different genotypes and color circles represent the zones

Most of the genotypes were identified in the eastern districts of Qom city in the neighborhoods that have middle or low levels of income. Seven areas in which most of the genotypes were identified were named as “zones” (Fig. 3). The largest zone in Qom is located in the northwest of Qom and was named as Z1, and the smallest is located in the southwest of the city and was named as Z7. While some genotypes including Delhi/CAS and NEW-1 were almost identified in all zones, the remaining genotypes were identified only in some zones. LAM was identified in Z3, Z4, Z5 and Z6, UgandaII in Z3 and Z7, Beijing in Z1 and Z2, EAI in Z7 and S in Z5.

### Allelic diversity of different loci

The allelic diversity (h) for each locus is shown in (Table 2). QUB4156 locus had the greatest diversity with h = 0.87 for 8 alleles. Thirteen MIRU loci were regarded as highly diverse with h > 0.6; ETRA, Mtub34, ETRC, ETRB, MIRU39, Mtub21, MIRU16, MIRU10, MIRU31, Mtub04, QUB26, MIRU26 and QUB4156), 8 were considered as moderately diverse with 0.3 ≤ h ≤ 0.6; Mtub30, QUB11b, Mtub29, MIRU40, MIRU27, MIRU23, MIRU20 and Mtub39), and the other MIRU loci (MIRU02, 04 and 24) had low levels of allelic variability (h < 0.3).

**Table 2:** Locus variability among the isolates examined

Locus	QUB4156	MIRU26	QUB26	Mtub04	MIRU31	MIRU10	MIRU16	Mtub21	MIRU39	ETRB	ETRC	Mtub34	ETRA	Mtub39	MIRU20	MIRU23	MIRU27	MIRU40	Mtub29	QUB11b	Mtub30	MIRU24	MIRU04	MIRU02
h	0.87	0.85	0.83	0.8	0.8	0.77	0.76	0.75	0.71	0.69	0.67	0.67	0.62	0.6	0.58	0.57	0.57	0.53	0.48	0.42	0.3	0.17	0.15	0.14

## Discussion

Before the COVID-19 pandemic, TB was considered as the first cause of death caused by an infectious agent in the world. The World Health Organization (WHO) has estimated that only in 2020, more than 1,500,000 people died of TB around the world [20]. This organization estimated that more than a third of all cases of active TB in the world are not diagnosed and are not reported to health centers. According to WHO, the COVID-19 pandemic caused a significant decrease in the diagnosis of new TB cases all over the world. The most immediate consequence of this large reduction in diagnosis, was the increase in TB deaths at all national, regional and global levels. Besides, the COVID-19 pandemic has reversed the result of several years of continuous effort to reduce the number of TB deaths from 2005 [21].

Several studies from different parts of the world have introduced immigration as one of the most important risk factors for TB [1–8]. Immigrants may contract TB during their residence in their native lands or even after immigration. As a result of the unfavorable economic and social conditions that immigrants might face, LTBI can turn into active disease. In Qom province, about 10% of the population are immigrants; immigrants who often arrive from neighboring countries such as Afghanistan and Pakistan [12]. The incidence of TB in these countries is higher compared to Iran [10]. Therefore, it was important to assess the impact of immigration on TB situation in Qom.

Thousands of immigrants come to Qom to study, practice and teach the Islamic directives. Normally, the classes of religion are held in groups including students from different nationalities that study together for several years. This might increase the risk of TB infection among these immigrants and the native students as well. In this study, the main genotypes among Afghan immigrants were Delhi/CAS, NEW-1 and Beijing. Also *M. tuberculosis* genotypes were identified in 3 other immigrants; a Pakistani clergy with NEW-1, an Indian clergy with LAM and an Indian housewife who harbored a MTB isolate that did not match with profiles present in MIRUVNTR*plus* database. The 3 genotypes belong to immigrants who lived in the same district that surrounds the holy shrine and other several Islamic schools.

Considering both the geographic distribution and the distribution over the time of genotypes show that among included isolates, there was a dominance of the two genotypes of Delhi/CAS and NEW-1. This might imply that immigrants and natives are spreading these genotypes among each other. The comparison of the present study with previous studies [22–25], indicate that NEW-1 and Delhi/CAS genotypes might be the most identified genotypes among Iranian TB patients; which is attributed to the living of Afghan immigrants in the country.

The only study that have mentioned *M. tuberculosis* genotypes in Qom province have been conducted 10 years ago using spoligotyping. This study showed that out of 61 isolates examined in Qom province, 20 isolates had Haarlem genotype, 18 had Delhi/CAS genotype, 10 Beijing genotype, 8 T genotype, 3 isolates that did not match with profiles present in MIRUVNTR*plus* database and 2 isolate with Bovis genotype [24]. That study lacked the demographic information of the patients and did not reported the risk factors of TB. Also, unlike the present study, the impact of immigration on TB transmission was not included. Therefore and in order to fill the existing informational gaps, the present study was done. In 2019, a study on the multidrug resistant (MDR) isolates recovered from Afghan immigrants, reported Haarlem (33.5%), Beijing (20.5%), Delhi/CAS (12.1%) and EAI (8.3%) genotypes [25]. In addition, they reported that of 76% of the isolates that showed the Beijing genotype were found among Afghan immigrants; but in the present study, Beijing genotype was only identified among Afghan immigrants. But the identification of this genotype exclusively in Afghan immigrants in comparison with different genotypes in natives didn’t reach a statistical significance. In 2015, a study showed that the frequency of Beijing and NEW-1 genotypes in the west and northwest of Iran is increasing due to the flow of Afghan immigrants [26]. Regarding the NEW-1 genotype, a research of Feyisa et al. in 2015 showed that the immigration of Afghans to Tehran province has increased the prevalence of NEW-1 genotype in Tehran [23]. In the present study, the identification of NEW-1 genotype might imply a similar fact. Also, S genotype was observed only in an isolate belonging to an Iranian patient. S, is a genotype that is not often observed in the country and is usually identified in Iran’s neighbor countries [11, 27–29].

Assessing the associations of genotypes with different risk factors showed that none of the risk factors were related to a specific genotype (P-value > 0.05). In housewives, which are the largest occupational group in this study, there was no dominant genotype and most of them were illiterate elderlies that had no previous contact with TB patients. Some reasons that likely have increased TB infection in elderlies are low education levels, lack of knowledge about the symptoms of TB and the route of transmission, lack of knowledge about follow-up methods for TB treatment, poverty and concern about the cost of treatment (some patients might thought that they have to pay for their treatment themselves while it is actually free). In laborers, Delhi/CAS genotype was the dominant genotype. Most of them were also Afghan elderlies and a third of the laborers had previous contact with TB patients.

Over the period of 5 years, Delhi/CAS and NEW-1 remained as the dominant genotypes and were identified almost in all zones in both natives and immigrants. Other genotypes which had lower frequency were identified exclusively in some zones. From these patterns of distribution, one can conclude that immigrants and natives are consistently transmitting Delhi/CAS and NEW-1 genotypes to each other as these genotypes were the dominant genotypes among all the included MTB isolates over the time.

## Conclusion

In conclusion, this study showed how immigration and its different reasons such as enrolling for religious studies and similarities in culture can have an impact on the genotypic diversity of *M. tuberculosis* in an area over the time. This study included information about the circulating *M. tuberculosis* genotypes, their geographical distribution and the association of TB risk factors with these genotypes and the impact of immigration on the situation of TB in Qom province.

## Electronic supplementary material

Below is the link to the electronic supplementary material.

Supplementary Material 1

## Data Availability

The raw data of this study was included in a supplementary file Table S1.

## List of Abbreviations

CTAB: Cetyl trimethylammonium bromide
LTBI: Latent tuberculosis infection
LJ: Lowenstein-Jensen
MTB: *Mycobacterium tuberculosis*
MTBC: *Mycobacterium tuberculosis* complex
TB: Tuberculosis
